# Supplementary material for: Emergence of coherent backscattering from sparse and finite disordered media
Source: Sci Rep. 2022 Dec 23;12:22256. doi: 10.1038/s41598-022-25465-y (PMC9789089; doi:10.1038/s41598-022-25465-y)
Supplement: Supplementary file 1 — Supplementary Information 1. [file 41598_2022_25465_MOESM1_ESM.pdf]

Supplemental Information for

"Emergence of coherent backscattering from sparse and finite disordered media"

Nooshin M. Estakhri[1,2]*, Nasim Mohammadi Estakhri[3], and Theodore B. Norris[2]

[1]Department of Physics, Virginia Tech, Blacksburg, Virginia 24061, USA
[2]Department of Electrical Engineering and Computer Science, University of Michigan, Ann Arbor, Michigan 48109, USA
[3]Fowler School of Engineering, Chapman University, Orange, CA 92866, USA
**estakhri@umich.edu*

This supplemental document includes the following two sections:

S1- More details on the formalism and numerical modeling.

S2- To what extent can effective quantities be useful in characterizing sparse wavelength-sized samples in scalar analyses?

S1- More details on the formalism and numerical modeling:

In this section we provide further details, complementary to the "Formalism and modeling" section in the main text, on the approach employed in this work for solving the Lippmann-Schwinger equation and calculating the scattering amplitudes for the individual and ensemble-averaged scattering intensities. We note that our approach is closely related to the discrete dipole approximation (DDA) which is also referred to as the coupled-dipole method in the literature.

DDA is an approach that is generally used for numerical simulation of electromagnetic scattering from particles with arbitrary shapes [S1], originally proposed by Purcell and Pennypacker [S2]. In this method a general scatterer (such as sphere or a structure with a complicated and asymmetric geometry) is spatially discretized and the scattering response from the initial geometry is approximated by the scattering response from the collection of point dipoles typically positioned at the center of the discretization grids. By finding a self-consistent solution to the Maxwell's equations for the collection of the electric dipoles, dipole polarizations are found from which the electric fields can be calculated everywhere. Ref [S1] provides a detailed historical review on this topic as well as a study over the development of this method. Here we follow a similar approach, but for the Lippmann-Schwinger equation, which comparably gives rise to solving a system of linear equations. This method can be understood as a scalar adaptation to the DDA method.

The scalar field $\mathrm{U}(\mathbf{r})$ satisfies the Lippmann-Schwinger integral equation given by Eq. (2) in the main text. For scattering from a collection of subwavelength spheres with radius "r", i.e., point-like scatterers, the susceptibility function can be replaced by a summation over bare polarizabilities of particles at the position of each scatterer, i.e., $\eta(\mathbf{r})=\sum_{\mathrm{i}=1}^{\mathrm{N_p}}\alpha_0\delta(\mathbf{r}-\mathbf{r}_\mathrm{i})$, in which $\alpha_0=\mathrm{r}^3\left(\mathrm{n}^2-1\right)/3$ [S3]. From this point, the Lippmann-Schwinger integral equation quickly reduces to a set of coupled equations for total scalar field at the position of the scatterers as

$$\mathrm{U}(\mathbf{r}_\mathrm{j})\left[1-\mathrm{k}_0^2\alpha_0\mathrm{G}_0(0,0)\right]=\mathrm{U}_\mathrm{i}(\mathbf{r}_\mathrm{j})+\mathrm{k}_0^2\alpha_0\sum_{\mathrm{i}=1,\mathrm{i}\neq\mathrm{j}}^{\mathrm{N_p}}\mathrm{G}_0(\mathbf{r}_\mathrm{j},\mathbf{r}_\mathrm{i})\mathrm{U}(\mathbf{r}_\mathrm{i})\cdot \qquad \text{(S1)}$$

Next, the Green's function has been properly regularized at $\mathbf{r}=\mathbf{r}'$ and the total field at the position of scatterers, $\mathrm{U}(\mathbf{r}')$, is found by direct inversion of the $\mathrm{N_p}\times\mathrm{N_p}$ matrix representing the above set of coupled equations. Besides the direct method, iterative methods can be applied to solve the set

of coupled equations such as the family of Krylov subspace methods. Such iterative methods are widely used in DDA approach [S4].

To find the scattered field in the far field, i.e., $|\mathbf{r}| \gg |\mathbf{r}'|$, we note that the free space Green's function behaves asymptotically as $G_0(\mathbf{r},\mathbf{r}') \sim e^{ik_0|\mathbf{r}|}e^{-ik_0\hat{\mathbf{r}}\cdot\mathbf{r}'}/|\mathbf{r}|$. This makes it possible to extract the outgoing spherical part from the scattering fields and find the total scattered field as

$$U_s(\mathbf{r}) \sim k_0^2 \frac{e^{ik_0|\mathbf{r}|}}{|\mathbf{r}|} \int d^3\,|\,\mathbf{r}'\,| e^{-ik_0\hat{\mathbf{r}}\cdot\mathbf{r}'} \eta(\mathbf{r}') U(\mathbf{r}') \cdot \qquad \text{(S2)}$$

Given that the total fields at the position of the scatterers, $U(\mathbf{r}')$, are already calculated in the previous step, the scattering fields can be then calculated in the far field. All the numerical results presented in the main text have been calculated using this approach.

S2- To what extent can effective quantities be useful in characterizing sparse wavelength-sized samples in scalar analyses?

In this section we take a look at some of the average quantities widely used in CBS studies to understand their implications for small finite-sample characterization. Previous theoretical efforts for studying CBS and solving the radiative transfer equation following the diffusion approximation, have led to approximate formulas for several average quantities concerning the CBS effect. These equations (e.g., see Eq. (3) in the main text) are valid for systems treatable as a composition of individual scatterers, i.e., with diffusion, approximation corresponding to the weak disorder limit of $kl \gg 1$. As shown in Fig. 5 of the main text, the CBS "line shape" for scattering from our spare wavelength-scale disordered structures can still be closely captured by such analytical predictions as given in Eq. (3) of the main text, that are originally found for diffusive

limits and under diffusion approximation. However, they don't capture the full picture, as discussed below. It is interesting to test, besides the line shape, another average quantity, namely the elastic scattering length. For this purpose, we first calculate the total scattering cross section using $\sigma = 4\pi k_0^4 r^6 (n^2-1)^2/9$ [S5] to be around $0.55\lambda_0^2$. Using the total cross section in combination with the geometric cross section and particle volume fraction, the elastic scattering length can be found to be around $l \approx 14.48\lambda_0$ (see e.g., [S6]). As such, the diffusive limit assumption [S5] considered in the theoretical derivations is no longer valid since the optical depth (L/l) is not much larger than one, especially for samples with sizes of $10\lambda_0$ or $20\lambda_0$. Therefore, it is important to conclude that the line shape still follows the theoretical form derived for large samples but the width of CBS cone for such small geometries can no longer be accurately extracted from the theoretical average quantities. Notably, as mentioned in the main text, the CBS cones broaden due to the finiteness of the sample, which is also the case here. If we continue to use theoretical average quantities under the diffusive limit, the cone width is approximated at 0.007 rad [S7]. This is about 20 times narrower than the width observed in the exact simulations and the broadening is the direct consequence of the wavelength-size dimensionally of the sample in all three directions. Thus, it is important to properly distinguish the CBS effect observed in large samples, which have been often the subject of experiments, with the enhancement from the structures under the study in this work.

Data availability

Data that supports the findings of this study are available from the corresponding author upon reasonable request.

References

S1. M. A. Yurkin, and A. G. Hoekstra, "The discrete dipole approximation: an overview and recent developments," J Quant. Spectrosc. Radiat. Transf. 106(1-3), 558-589 (2007).

S2. E. M. Purcell, and C. R. Pennypacker, "Scattering and absorption of light by nonspherical dielectric grains," Astrophys. J. 186, 705-714 (1973).

S3. J. C. Schotland, A. Cazé, and T B. Norris, "Scattering of entangled two-photon states," Opt. Lett. 41(3), 444-447 (2016).

S4. B. T. Draine, and P. J. Flatau, "User guide for the discrete dipole approximation code DDSCAT 7.3," arXiv preprint arXiv:1305.6497 (2013).

S5. E. Akkermans and G. Montambaux, Mesoscopic physics of electrons and photons (Cambridge university press, 2007).

S6. O. Mengual, G. Meunier, I. Cayré, K. Puech, and P. Snabre, "TURBISCAN MA 2000: multiple light scattering measurement for concentrated emulsion and suspension instability analysis," Talanta 50(2) 445-456 (1999).

S7. Y. L. Kim, Y. Liu, V. M. Turzhitsky, H. K. Roy, R. K. Wali, H. Subramanian, P. Pradhan, and V. Backman, "Low-coherence enhanced backscattering: review of principles and applications for colon cancer screening," J. Biomed. Opt. 11(4), 041125 (2006).
